# Experiences and Perspectives of Marketing Authorisation Holders towards Medication Safety Monitoring during Pregnancy: A Pan-European Qualitative Analysis

**DOI:** 10.3390/ijerph19074248

**Published:** 2022-04-02

**Authors:** Laure Sillis, Veerle Foulon, Jan Y. Verbakel, Michael Ceulemans

**Affiliations:** 1 Clinical Pharmacology and Pharmacotherapy, Department of Pharmaceutical and Pharmacological Sciences, KU Leuven, 3000 Leuven, Belgium; laure.sillis@kuleuven.be (L.S.); veerle.foulon@kuleuven.be (V.F.); 2 Department of Public Health and Primary Care, KU Leuven, 3000 Leuven, Belgium; jan.verbakel@kuleuven.be; 3 Nuffield Department of Primary Care Health Sciences, University of Oxford, Oxford OX2 6GG, UK; 4 L-C&Y, KU Leuven Child & Youth Institute, KU Leuven, 3000 Leuven, Belgium

**Keywords:** pregnancy, medicines, drug information, drug safety, pharmacovigilance, pharmacoepidemiology, pharmaceutical industry, stakeholders, healthcare professional, healthcare

## Abstract

Although marketing authorisation holders (MAHs) are involved in monitoring medication safety, it was unclear how they experience their role and current monitoring activities in pregnancy. Therefore, a qualitative study using online focus groups with MAHs and the Belgian umbrella organisation of MAHs was conducted in June–July 2021. In total, 38 representatives of nine organisations participated. Overall, participants reported multiple difficulties with data collection, including underreporting, collection of incomplete information, and loss to follow-up. The limited number of high-quality data collected, the unknown denominator and the lack of comparator data complicate MAHs’ data processing activities, preventing them to timely provide evidence in the pregnancy label. Three ‘conflicts’ inherent to the specific position of MAHs were identified explaining the difficulties they experience, i.e., (1) mistrust from patients and healthcare professionals (HCPs); (2) MAHs’ legal obligations and regulatory framework; (3) MAHs’ position outside the healthcare context. To overcome these barriers, MAHs suggested that data registration should occur in close collaboration with patients and HCPs, organised within the healthcare context and performed by using a user-friendly system. In conclusion, the reported difficulties and underlying conflicts of MAHs highlight the need for more effective, collaborative data collection strategies to generate new evidence on this topic.

## 1. Introduction

Medication use during pregnancy is very common and may have increased over the last decades [1]. Currently, it is anticipated that more than 80% of women living in Europe use at least one medication during pregnancy [2]. However, there is a substantial need for more evidence on the safe use of medication during this critical period. Out of 172 medicines approved by the Food and Drug Administration (FDA) between 2000 and 2010, 98% was missing clear information on the teratogenic risk of the product [3]. The overall lack of data required to correctly estimate the fetal risk by medicines was confirmed by another study showing that good to excellent safety data were only available for 4% of a list of medicines commonly used by pregnant women [4]. In general, almost all medicines enter the market without having (sufficient) information on the risk of birth defects or adverse effects in the offspring. Moreover, it was shown that on average 27 years are needed to assign a medication with an ‘undetermined’ teratogenic risk to a more precise risk category [3]. Thus, it may take decades before teratogenic effects are identified, clearly revealing a serious public health problem [5].

One of the reasons behind this paucity of safety information is the exclusion of pregnant women from clinical trials due to ethical considerations. A review published in 2013 showed that only 1% of phase IV trials sponsored by pharmaceutical industry were designed specifically for pregnant women, while 95% of these trials excluded pregnant women [6], and this was not expected to improve in the near future [7]. Unfortunately, in recent history, the initial COVID-19 vaccine trials also excluded pregnant women, preventing women to benefit from the advantages of vaccination [8,9]. In the absence of data from human studies, one can only rely on pre-clinical and/or animal data, although there are limitations with respect to the extrapolation of animal data to humans [10,11,12].

Obviously, marketing authorisation holders (MAHs) play a key role in obtaining and enhancing the knowledge on medication safety during pregnancy, including providing the most recent information in the products’ label. In general, MAHs collect observational, ‘real-world’ data on medication safety during pregnancy through their post-marketing spontaneous reporting or surveillance systems. Sometimes, MAH set-up and manage product-specific registries, which might be imposed by regulators as part of post-approval requirements [13]. Finally, international existing databases such as EUROmediCAT and VigiBase containing individual case safety reports (ICSRs) could be used by MAHs for signal detection of congenital anomalies [14]. However, there is neither collection of control data in these databases, nor compilation of adverse effects of medication use during pregnancy such as miscarriage, stillbirth, growth restriction, pre-term birth, or neonatal withdrawal symptoms [14].

To support MAHs in their responsibility to monitor medication safety in pregnant and breastfeeding women, the European Medicines Agency (EMA) and FDA developed (draft) guidelines on registry-based studies and good pharmacovigilance practice (GVP) for medicinal products used in this population [15,16,17]. The latter two guidelines were still under revision when this article was written, and questions remain whether enough operational details are provided to substantially improve pharmacovigilance practice for MAHs [18]. Although there is evidence on the paucity of data in the labels [3,4], it is generally unknown how MAHs experience their current safety monitoring activities themselves and how they reflect upon their responsibilities, underlining the research gap and opportunities to investigate the perspective of MAHs in this regard. Such insight can contribute to identifying remedies tackling current difficulties or barriers and/or lead to new, more successful strategies to enhance the knowledge on medication safety in pregnancy. Therefore, this study aimed to gain insight into the current initiatives, needs, obstacles, expectations, and future preferences of MAHs regarding medication safety and pharmacovigilance in pregnancy.

## 2. Methods

### 2.1. Study Design

A qualitative study using online focus group discussions with MAHs was performed from June to July 2021. Focus groups were organised until data saturation was reached. Ethical and privacy approval was obtained from the Social and Societal Ethics Committee of KU Leuven (G-2021-3245; 23 April 2021). All participants provided electronic informed consent prior to study enrolment, while written approval for the participation of the employees in the group discussion was obtained from the responsible manager of the respective organisations. This manuscript is written in accordance with the COREQ guideline (see Table S1) [19].

### 2.2. Study Population and Sampling

The study population consisted of Dutch and English-speaking employees of MAHS working at national (i.e., Belgian) or global departments, and employees of the Belgian umbrella organisation of pharmaceutical companies. Employees of MAHs were eligible for participation if their current professional activities were closely related to medication safety and pharmacovigilance in pregnancy, and if their company (i.e., responsible manager) agreed to participation of the company in this study. A purposive sample technique was applied to contact MAHs with at least one department in Belgium, ensuring the inclusion of companies with different characteristics in terms of portfolio and areas/diseases of interest. Local contact persons within each company were approached and received the study information by e-mail. They were asked to share the invitation with their responsible manager and subsequently with their international colleagues in the company, and to specifically contact colleagues working at departments of pharmacovigilance, medical affairs, and pharmacoepidemiology. Since the organisation among companies may vary, employees affiliated to other departments whose work is closely related to the topic of interest could also be invited. Only one focus group for each organisation was organised.

### 2.3. Data Collection

The focus groups were structured using a topic guide consisting of open questions to explore current initiatives, needs, obstacles, expectations, and future preferences of MAHs regarding medication safety and pharmacovigilance in pregnancy. The topic guide was pilot tested in one organisation and modified accordingly. All focus groups were moderated by researchers M.C. (PhD, PharmD; post-doctoral researcher ‘medication use during pregnancy’; male) and L.S. (PharmD; PhD candidate ‘medication use during pregnancy’; female); both had experience in conducting (online) focus group discussions. Each focus group started with a personal introduction of the participants, including the moderators and their research topic, and with a description of the study objectives and structure of the focus group. The expected duration of a focus group was estimated to be about one hour and one hour 30 min. No repeat interviews were carried out. M.C. and L.S. took handwritten notes during the conversations. Focus groups were audiotaped and subsequently transcribed verbatim, while removing any personal data that could identify participants or organisations. Neither transcripts nor findings were returned to participants for corrections or additions. Audiotapes were destroyed after transcriptions were made.

### 2.4. Data Analysis

The transcripts were analysed by performing an inductive thematic analysis according to the framework method by Gale et al. [20]. To familiarise themselves with the data, transcripts were read and reread several times by L.S. and M.C. Using pen and paper, inductive coding was applied for the first four interviews. Intermediate discussions with the entire research team were arranged to compare codes and reflect upon the preliminary coding framework. The discussions resulted in an analytical framework that was applied to the other interviews, mindful that codes could be modified until the last interview was coded. NVivo software (release 1.5.1) was used as software [21].

## 3. Results

### 3.1. Characteristics of the Study Participants

In total, the researchers invited 19 different MAHs and the Belgian umbrella organisation of MAHs. Overall, online focus group discussions were held with 38 representatives of nine different organisations (eight MAHs and one umbrella organisation). The discussions lasted between one hour and one hour 45 min, with a mean duration of 87 min. The number of participants within each focus group varied between two and six, with a mean number of four. The mean age of participants was 47 years (range: 31–66), while most of them were working in pharmacovigilance departments in Belgium or the USA. Demographics of the participants and information about the organisations are shown in Table 1.

The results in the analytic framework (coding tree) were structured around three different domains: the collection of data on medication use during pregnancy (Section 3.2), the processing of these data into new evidence (Section 3.3), and the communication in the label (Section 3.4).

### 3.2. Collection of Data on Medication Use during Pregnancy

#### 3.2.1. Difficulties with Data Collection on Medication Use during Pregnancy

**Figure 1:**
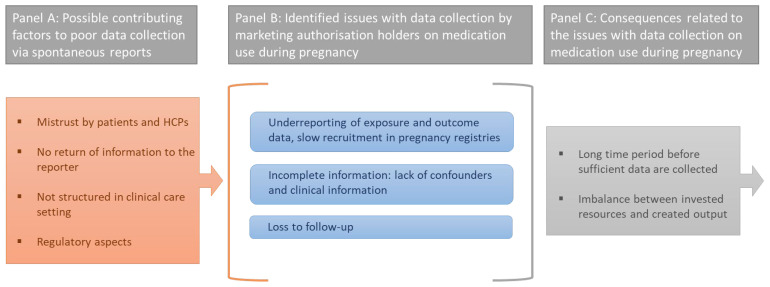
Overview of the identified issues (centre), possible contributing factors (left panel), and consequences related to data collection on medication use during pregnancy (right panel).

Participants confirmed that companies strongly rely on post-marketing spontaneous reports to obtain data on medication use during pregnancy, especially as pregnant persons are excluded from the standard clinical trials. However, all participants jointly acknowledged that companies struggle with collecting spontaneously reported exposure and outcome data related to medication use during pregnancy. Several issues were hereby identified by the participants, along with possible contributing factors and consequences of these issues related to data collection (Figure 1).

As a first issue, participants indicated that underreporting of exposure and outcome data both by patients and healthcare professionals (HCPs) is a major problem. Underreporting does not only occur for routinely collected spontaneous reports but also for medication for which pregnancy registries are in place. In fact, it was even stated that, for registries, underreporting is a larger problem for medication exposures that eventually do not result in adverse outcomes.

*There is a big black hole of missing information. It is not because exposures are not happening, it is because we are not able to collect them efficiently. [IT06-PP04]*

*For 2020, we got approximately 90 reports of pregnancy exposure worldwide. This out of an estimated exposure of roughly half a million patients worldwide. [IT07-PP03]*

A second issue that was raised by participants related to their struggle to obtain complete information about specific cases. Some employees stated they were unable to succeed in collecting sufficient details on exposure or relevant background, or mentioned difficulties with obtaining confirmations by HCPs. As a result, information on relevant variables and potential confounders remains unknown. Furthermore, a high percentage of loss to follow-up (LFU) was raised by interviewees as a common issue leading to a lack of outcome data.

An important contributing factor to poor data collection via spontaneous reports, which was often brought up by the interviewees, was mistrust by patients and HCPs towards the surveillance activities carried out by MAHs. Participants answered having the impression that they were lacking credibility in the eyes of patients and HCPs, which negatively affected the data collection process.

*One of the biggest barriers to overcome is a sense of mistrust. [IT09-PP01]*

Other participants expressed discomfort by not having the opportunity to directly return information to the reporter. They felt their position was only requesting data and time from reporters with nothing in return. Additionally, participants mentioned that their surveillance activities are not embedded in clinical care settings, preventing them from close interactions with reporters. Moreover, participants stated running into barriers related to regulatory aspects. First, MAHs expressed difficulties with obtaining the obligatory consent from participants, which is required a priori to allow follow-up. Second, they indicated experiencing difficulties with the specific regulatory guidelines applicable to pregnancy registries, i.e., pregnancy registries come with limitations on how patients can be sampled (e.g., strict rules for advertising), creating challenges to obtain a sufficiently large number of inclusions.

*We have rather none, or very little experience on sending out the questionnaires on pregnancy outcomes because the process usually stops at the stage of the consent (after receiving the notification of an exposure during pregnancy). It is a 7-page document that is actually saying: we only want something from you and you do not get anything from us. We do not get these signed pages back… [IT08-PP01]*

As a result of the underreporting and collection of incomplete exposure and/or outcome data, interviewees stated that it takes a very long time before sufficient data are collected to correctly estimate the teratogenic risk of a medication (thereby assuming that the quality of the collected data is sufficient). In the meantime, safety concerns related to this (new) medication remain.

*We could have a drug that is relatively new on the market, which passes all the clinical trial stages, but never really being used in a pregnant woman. The limitation of this spontaneous reporting system is that it may delay the detection of a potential issue. (…) In the spontaneous reporting system, it will take a while before we reach the evidence required to really draw conclusions: the poorer the quality, the slower the report coming in, the longer it will take until someone says, ’wait a second, I have a bad feeling here‘. [IT07-PP03]*

Moreover, employees stated that companies are confronted with an imbalance between the resources invested in activities related to medication safety in pregnancy versus the output created by the current procedures or approach. Non-successes and dissatisfactions with company-initiated pregnancy registries were mentioned several times.

*After 5 years of running the registry, we had no reported exposed pregnancies. So, it was a lot of effort and a lot of money [invested] by the company, and at the end of the day we did not yield any useful information. [IT08-PP03]*

*I don’t mind having to do all sorts of work and invest time in a pregnancy registry. My issue with that is, still after all these resources and time, many interim assessments, you still don’t have a handle on how safe it is. [IT09-PP03]*

#### 3.2.2. Data Collection on Long-Term Outcomes Related to Pre-Natal Medication Exposure

Data collection on long-term outcomes after pre-natal medication exposure was cited by many interviewees as an important and relevant area, but many considered it to be a very complicated task for MAHs. The previously mentioned difficulties related to conducting follow-up directly after birth are only larger for long-term follow-up given its greater need for involvement in the care context and associated trust. To make it feasible, one participant reported that this should rather be initiated on an academic level.

*It is really important that long-term data collection is done, but it should be pursued from an academic rather than from a company perspective, even though it is important for the company. [IT04-PP02]*

#### 3.2.3. Suggestions to Improve Data Collection on Medication Safety during Pregnancy

Some participants suggested that an increased connection between reporter (patient or HCP) and data collector may facilitate data collection practice. This could include being involved in the HCP-network as a data collector to optimise reporting and detailing by HCPs, but also engaging in actual conversations with patients when reporting exposure. However, some interviewees elaborated on difficulties they experience to become involved in the HCP-network and felt that professionals present in the healthcare context of pregnant persons might be better placed to fulfil this task than employees of MAHs. This was in line with experiences of some participants regarding patient support programs where companies are more involved in the local healthcare context and where data collection may be more successful, especially in case of uncomplicated pregnancies.

*Maybe we can encourage the reportingofpatients not by directly addressing the patients by us as MAH, but* via *the channels which have a direct link with pregnant women, who follow them up during pregnancy and in the first years after birth? For example, Birth and Childhood Offices? [IT09-PP02]*

*Following-up with somebody who knows the local medical healthcare context and the patient. I (as an MAH) can’t do that. (…) We are collecting hugely sensitive data, it takes a real level of trust to be able to do that and to do it in a good way. [IT04-PP02]*

Concerning the initiation of data collection, some participants suggested that organisations other than MAHs would be more appropriate and/or successful. A main reason therefore was that independent organisations would be more trusted by patients and HCPs compared to MAHs. Another suggestion that was often brought up by the interviewees was the importance of the initiation/implementation of disease-specific registries instead of product-specific registries (‘at the company-level’) given the advantages for data analysis (see further). However, some participants pointed out that potential reluctance among MAHs might be present to (voluntarily) start collaborations with a potential competitor.

*Often, we do not have the credibility, there is a lot of hesitancy. An academic institution adds also lot of credibility to the data that comes out of it. [IT08-PP03]*

*We should look to partner more with agencies and groups where people feel more comfortable sharing information with, than potentially a company. [IT09-PP01]*

*If a company sets up a registry, it is product-based, while in fact scientifically speaking it is better to set up disease-specific registries. [IT01-PP02]*

Other participants stated that improvements could be made regarding the operational part of data collection, i.e., making it easier for patients and HCPs to report, decreasing the administrative burden. Some participants mentioned the introduction of mobile apps, for example, to facilitate reporting by patients.

*I think one of the answers [to how* *administrative* *burden can be decreased] would be, to use a mobile app. Everyone has a phone, right? I think this is the direction in which we are all going, whether we like it or not, to move towards more a digital era. [IT04-PP06]*

Given the difficulties and challenges for MAHs with collecting data through spontaneous reporting, participants also reflected upon alternative methods to obtain evidence on medication safety in pregnancy. For example, the secondary use of data collected in electronic health records and administrative databases spontaneously came up during the discussions. Participants saw the availability of big data in these databases as the main advantage. However, secondary use of data also comes with limitations, for example, as the fragmentation of data sources. Although secondary use of data could be an interesting alternative method, interviewees felt that its effectiveness depends on the combination of multiple data sources, including spontaneous reporting and (disease-based) registries. The importance of a holistic approach to generate new evidence on medication safety in pregnancy was highlighted by other participants.

*To tackle our issues with slow* *recruitment* *, it would help to combine different sources of evidence: pharmacovigilance data, and secondary use of data; all in one protocol in one package. [IT05-PP01]*

*No piece alone will be perfect, but* *together* *we can make a better effort. [IT04-PP02]*

### 3.3. Processing of Observational Data on Medication Use during Pregnancy

Overall, participants mentioned several challenges when trying to process the data into new evidence. These challenges were quite similar to the previously mentioned difficulties related to data collection. First, due to the underreporting, participants highlighted that the number of spontaneously collected reports is too small to reliably answer questions regarding potential risks. Second, the absence of a correct denominator, along with the likelihood of an overreporting of cases with adverse outcomes, was considered a real challenge when analysing the data, resulting in incorrect risk estimates. Third, participants stated that the quality of reports was often very poor and insufficient with regard to relevant confounders (e.g., maternal age or comorbidities) and timing of exposure. Finally, the absence of comparator data in a disease-matched, non-exposed population was another major concern.

*From the time that the* *information* *is available, I can tell you, it is a nightmare. We cannot really get a hold of the data, we conduct studies, but most of the times the studies have a lot of shortcomings. [IT02-PP04]*

*The quality and* *completeness* *of the information we have is very often extremely poor and does not really allow an accurate assessment. [IT07-PP03]*

### 3.4. Communication of Safety Information in the Label 

Most participants acknowledged that MAHs do not have sufficient data on medication safety during pregnancy to provide decisive statements for the pregnancy section in the label or Summary of Product Characteristics (SmPC). According to the interviewees, this is the result of the abovementioned difficulties with data collection and processing, and not because of MAHs unwillingness to include the available data in the label.

*There is perception, I think, from general public and even sometimes from practicing physicians, that the data is there and we’re just not putting it in the label or making generally available. The truth is: methodologies are not terribly efficient yet to gather this type of* *data* *. [IT06-PP04]*

Some participants acknowledged that the pregnancy section in the label often only includes a ‘vague’ statement and some pre-clinical information, which could have multiple severe implications in practice. First, lack of clear information in the label may put HCPs and patients in a very difficult position to make a decision without being able to adequately balance risks and benefits.

*Most of the time we don’t have* *enough* *information to state anything defined in the label. Instead you do see some vague statement, like ‘has not been studied’. [IT02-PP06]*

*To me, that’s a patient group that is currently left to its own devices. It’s really only the individual decision of the health care provider whether they will continue with that treatment or not. (…) Nobody wants to run the liability to give the advice: ‘just do it, it will* *be* *fine’; you can’t do that if there is no data. [IT01-PP01]*

Another potential implication, as mentioned by some participants, was that this lack of information in the label may deprive patients from medications from which they could actually benefit.

*I think by not studying [* *medications* *in pregnant women] and not having enough information in the label, we might deprive pregnant women who would actually benefit from a drug with a good benefit-risk profile. [IT02-PP06]*

Concerning the label, uncertainties were expressed towards the regulatory context of changing the pregnancy information. Participants mentioned that it is clear for companies how they should list signals or adverse events in the label, but they experience difficulties with providing statements on the safe use during pregnancy in the label. Furthermore, dissatisfactions with both the infrequent changes and the slow process of changing labels were mentioned multiple times. Participants attributed this respectively to the paucity of data to update the label and ponderous regulatory processes of changing them. Some interviewees raised safety implications of these infrequent and slow changes, such as not being able to timely communicate on precautions when needed. Finally, some participants also reflected upon the fact that the label is a legal document that comes with liability implications, thereby expressing concerns related to the responsibility of MAHs in this regard.

*It is very clear what it* *takes* *to get a signal or an adverse event in the label. It is not as clear for us to know what it takes to say that it is safe to be used during pregnancy. (…) We don’t even know what we can do to get language in our label that says that our products are safe. [IT02-PP04]*

*The idea of giving* *medicines* *to pregnant women without sufficient safety assurance is like dynamite. We won’t touch it, we won’t touch it for the patient, we won’t touch it for the regulators and we won’t touch it because of the medical legal situation. [IT07-PP04]*

## 4. Discussion

### 4.1. Main Findings

**Figure 2:**
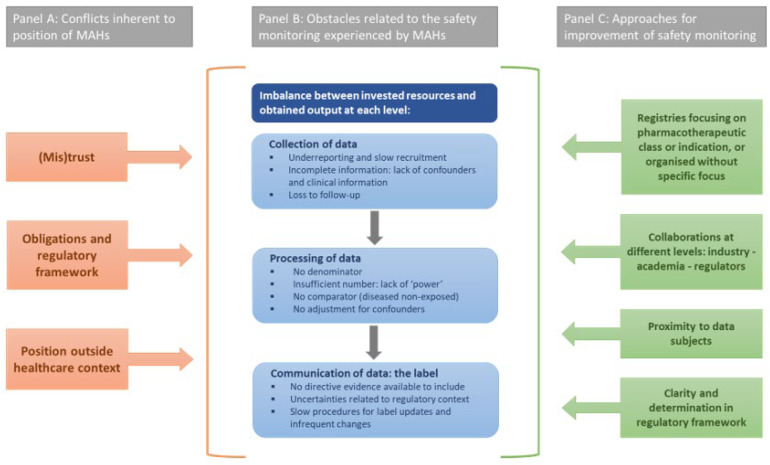
Overview of the obstacles related to the safety monitoring experienced by MAHs (**panel B**), including underlying conflicts (**panel A**) and approaches for future improvement (**panel C**).

This qualitative study using online focus groups and involving employees of MAHs working in Europe and North America aimed to provide insight into the experiences and perspectives of MAHs towards the monitoring of medication safety during pregnancy. The focus groups yielded an open, constructive, reflective, and future-oriented dialogue with representatives of eight MAHs and one national umbrella organisation. Overall, participants expressed their common disappointment with the poor return of their monitoring efforts in terms of increasing knowledge on medication safety in pregnancy. Participants did not minimise the issues they are struggling with related to this topic. Based on MAHs’ experiences with data collection, data processing, and communication of information in the label (see panel B, Figure 2), three ‘conflicts’ inherent to the position of MAHs were identified, each of them contributing to the obstacles observed in the three domains (see panel A, Figure 2).

The first ‘conflict’ relates to the concept of (mis)trust. While patients and HCPs are the main actors to provide data, participants indicated that both actors often show suspicion towards data reporting to MAHs. According to the interviewees, patients and HCPs have mixed feelings towards the role MAHs play in taking responsibility for the safety of their products. Patients and HCPs seem to find this role ambiguous and conflicting, especially given the commercial interests of companies. In addition, employees expressed concerns regarding data sharing with other MAHs as part of disease-specific registries, emphasising the competitiveness and mistrust within the pharmaceutical industry itself.

A second ‘conflict’ is related to the obligations and regulatory framework of MAHs. MAHs do have the obligation to perform pharmacovigilance activities and monitor medication safety. However, MAHs strongly indicated that the current monitoring activities related to medication use in pregnancy do not lead to the output or evidence that is desired and asked for by regulators, the public and HCPs. Next to these legal obligations, the regulatory framework implies strict rules and restrictions on how their responsibilities should translate into actions. MAHs indicated struggling with this regulatory framework as it hinders them to take up their role, for example, when trying to motivate patients to enrol in their registries or when collecting follow-up data. Both examples show that MAHs are in a feeble and conflicting position, illustrated by an imbalance between investing resources to fulfil their legal obligations and constraining factors inherent to the regulatory framework.

Finally, the last ‘conflict’ is about the context where data collection activities take place. The monitoring activities of MAHs are carried out at the company-level and are basically not structured within the healthcare setting. Although MAHs considered proximity to the healthcare setting, patients, and HCPs as an important prerequisite for successful data collection, they felt this is not feasible given their position as MAHs. Overall, these three ‘conflicts’ are inherent to the position of MAHs and explain the impasse between invested resources to fulfil the requirements of safety monitoring of medication use in pregnancy and the limited output obtained with regard to data collection, data processing, and communication via the SmPC label. More specifically, these findings uncover that MAHs are not the most effective actor to generate evidence on medication safety in pregnancy, and urge for collaborative and more effective strategies to collect sufficient (real-world) data on maternal medication use and mother–infant outcomes.

### 4.2. Approaches for Future Improvement of Safety Monitoring

During the focus groups, fragmentary ideas to improve safety monitoring practices were brought up by MAHs. These ideas, along with an in-depth understanding of the obstacles mentioned in the focus groups, allowed us to identify four approaches for future improvement (see panel C, Figure 2). First, instead of product-specific registries, registries should focus on a pharmacotherapeutic class [23] or a therapeutic indication [24,25], or be organised without any specific focus on medication or disease [26]. These registries were successful in the past. Thereby, academia and teratology information services have gained experience over the years on how exactly prospective registries at the population-level should be organised [26,27]. Second, a collaborative approach involving different stakeholders (i.e., pharmaceutical industry, regulators, and academia) needs to be established to prospectively collect exposure and control data [28,29,30]. Thereby, opportunities of combining multiple data sources should be exploited, as suggested previously [31,32,33,34]. As MAHs stated that it is difficult from their position to take the lead in these collaborations, it is suggested that regulators initiate and encourage collaborations. Efforts to align industry, academia, and regulators in this field are currently undertaken by the IMI ConcePTION project [22]. A third approach relates to the importance of proximity of and trust from reporters, notwithstanding establishing collaborations on international level. More specifically, data collection should be embedded in the local healthcare context with a strong connection to patients and HCPs [35,36]. Finally, MAHs suggested more determination and clarity in the applicable regulatory framework, including innovative and supportive guidance towards the three other approaches for future improvement. Yet, it remains a question to what extent these approaches will be tackled in the final version of the EMA and FDA guidelines that are currently under review.

### 4.3. Strengths and Limitations

The qualitative approach of this study involving both national and international employees of large and influential MAHs allowed an in-depth understanding of the current needs, obstacles, and future preferences of MAHs in Europe and North America regarding safety monitoring of medication use in pregnancy. Moreover, employees working at different departments (i.e., pharmacovigilance, epidemiology, medical affairs, regulatory affairs) and viewing this topic from different perspectives were included, broadening the insights derived from this study. Finally, the focus groups were held at an organisation-level bringing employees of the same organisation together, ensuring people to express their opinions and MAH-based experiences freely. Afterwards, this was considered a good choice as we felt some competition among MAHs. However, some limitations could also be addressed. First, the purposive sampling technique may have led to some selection bias. As five of the nine participating MAHs are involved in the ongoing IMI project ConcePTION, they may have a strong pre-existing interest in the topic of medication safety during pregnancy. Second, employees of different hierarchical levels participated in the same interview. Because of the hierarchical subordination, some employees may have felt restricted in expressing certain opinions, although we generally did not have this impression. Third, only a few MAHs with a focus on biotechnology were included in the sample. Finally, regulatory agencies were not interviewed as part of this study, and hence, their perspectives could not be compared with those of MAHs. As this study only focused on the perspectives of MAHs towards medication safety among pregnant women, which are considered a very specific population in terms of drug safety, pharmacovigilance, and participation in (clinical) trials, we believe that the findings cannot easily be extrapolated to other ‘vulnerable’ populations, although some similar obstacles may exist for spontaneous reporting and observational research using real-world data in other groups.

## 5. Conclusions

To enhance medication safety in pregnancy, a large number of complete and preferably prospective registrations of both exposed and non-exposed pregnancies are needed, including sufficient details on potential confounding factors. Unfortunately, MAHs jointly acknowledged experiencing multiple obstacles regarding data collection, processing, and communication of evidence in the label, perpetuating the existing lack of safety evidence on this topic. MAHs suggested that data registration should occur in close collaboration with patients and HCPs, organised within the healthcare context and performed by using a user-friendly system. This study further identified three ‘conflicts’ related to the specific role and position of MAHs explaining the obstacles they experience, i.e., a lack of trust from patients and HCPs, MAHs’ legal obligations and the regulatory framework, and MAHs’ position outside the healthcare context. These conflicts highlight the need for more effective, collaborative strategies to prospectively collect (real-world) data to generate new evidence and to fill the current information gap on medication safety in pregnancy.

## Figures and Tables

**Table 1:** Demographics of the participants and information about the organisations.

Demographics of Participants (N = 37) ^1^	
Gender	
Female	26 (70.3%)
Male	11 (29.7%)
Highest educational level	
Bachelor	2 (5.4%)
Master	19 (51.4%)
PhD	16 (43.2%)
Department of the current function	
Pharmacovigilance	19 (51.4%)
Medical affairs	6 (16.2%)
Epidemiology	4 (10.8%)
Regulatory affairs	2 (5.4%)
Other	6 (16.2%)
Location current function	
Belgium	15 (40.5%)
USA	11 (29.7%)
Other European countries	11 (29.7%)
**Information on the organisations (N = 9)**	
Departments in different countries	7 (77.8%)
Location headquarters	
USA	3 (33.3%)
Belgium	2 (22.2%)
Switzerland	2 (22.2%)
UK	1 (11.1%)
Japan	1 (11.1%)
Participation in IMI ConcePTION ^2^	5 (55.6%)

Results are shown as absolute numbers (%). ^1^ Information on the demographics of one participant is missing; ^2^ IMI ConcePTION is a public–private partnership launched in April 2019, aiming to build an ecosystem for medicine safety in pregnancy and breastfeeding [22].

## Supplementary Materials

The following supporting information can be downloaded at: https://www.mdpi.com/article/10.3390/ijerph19074248/s1, Table S1: COREQ checklist.
